# Pregnancy outcomes in women with pulmonary hypertension: a retrospective study in China

**DOI:** 10.1186/s12884-023-05353-7

**Published:** 2023-01-09

**Authors:** Chengtian Lv, Yuwen Huang, Guangyuan Liao, Lichan Wu, Dunjin Chen, Yuanmei Gao

**Affiliations:** 1 Department of Critical Care Medicine, Guangdong Provincial Key Laboratory of Major Obstetric Diseases, The Third Affiliated Hospital of Guangzhou Medical University, Guangzhou, China; 2 Department of Obstetrics and Gynecology, Department of Fetal Medicine and Prenatal Diagnosis, Guangdong Provincial Key Laboratory of Major Obstetric Diseases, The Third Affiliated Hospital of Guangzhou Medical University, Guangzhou, China

**Keywords:** Pregnancy, Pulmonary hypertension, Maternal-foetal outcome

## Abstract

**Background:**

In recent years, with the development of monitoring conditions and the application of pulmonary vascular-targeted drugs, pregnancy outcomes in women with pulmonary hypertension (PH) have improved, but the maternal mortality rate is still high. The purpose of this study was to describe the maternal-foetal outcomes in pregnant women with PH.

**Methods:**

The clinical data of 154 pregnant women with PH who were admitted to the Third Affiliated Hospital of Guangzhou Medical University from January 2011 to December 2020 were collected and descriptively analysed.

**Results:**

Among the 154 pregnant women with PH, 6 (3.9%) had idiopathic pulmonary arterial hypertension (iPAH), 41 (26.6%) had pulmonary arterial hypertension (PAH) associated with congenital heart disease (CHD-PAH), 45 (29.2%) had PAH related to other diseases (oPAH), and 62 (40.3%) had PH related to left heart disease (LHD-PH). The systolic pulmonary artery pressure (sPAP) was 36–49 mmHg in 53.2% of the patients, 50–69 mmHg in 22.1% of the patients and ≥ 70 mmHg in 24.7% of the patients. Five (3.2%) pregnant women died within 1 week after delivery; iPAH patients had the highest mortality rate (3/6, 50%). Fifty-four patients (35.1%) were admitted to the intensive care unit (ICU), and the incidence of heart failure during pregnancy was 14.9%. A total of 70.1% of the patients underwent caesarean section; 42.9% had premature infants; 28.6% had low-birth-weight (LBW) infants; 13.0% had very-low-birth-weight (VLBW) infants; 3.2% had extremely-low-birth-weight (ELBW) infants; 61% had small for gestational age (SGA) infants; and 1.9% experienced neonatal mortality.

**Conclusion:**

There were significant differences in the maternal-foetal outcomes in the iPAH, CHD-PAH, oPAH and LHD-PH groups. Maternal mortality was highest in the iPAH group; therefore, iPAH patients should be advised to prevent pregnancy. Standardized and multidiscipline-assisted maternal management is the key to improving maternal-foetal outcomes.

## Introduction

Pulmonary hypertension (PH) refers to a clinical and pathophysiological syndrome with the pulmonary vascular resistance and pulmonary arterial pressure increase due to changes in pulmonary vascular structure and function, which are caused by a variety of heterologous diseases (causes) and different pathogenic mechanisms. Patients with PH are at high risk of right heart failure and even death [1, 2]. The prevalence of PH in women is 97 cases per million [3, 4]. Although the incidence of PH in pregnancy is very low, the mortality rate of pregnant women and foetuses is extremely high. Some studies have reported that, previously, without advanced pulmonary vascular targeting drugs, the mortality rate of pregnant women with PH was as high as 30–56% [5, 6]. The preterm birth rate was 85–100%, and the foetal or neonatal loss rate was 7–13% [7, 8]. Pulmonary arterial hypertension (PAH) in pregnancy threatens the health of pregnant women and foetuses.

Women with PH have haemodynamic changes during pregnancy, such as an increased total blood volume, an increased cardiac output, a faster heart rate, reduced systemic vascular resistance, and hypercoagulable blood. These physiological changes increase the risk of refractory right heart failure, PH crisis, pulmonary embolism, and even death [9]. The European Society of Cardiology (ESC)/European Respiratory Society (ERS) guidelines on the diagnosis and treatment of PH have always recommended contraception or early termination of pregnancy [3, 7]. However, during clinical work, we still face many women with PH who choose pregnancy or experience unplanned pregnancies. Especially in recent years, with the change in our country’s family planning policy and individual choices, the number of elderly pregnant women and high-risk pregnant women has increased, which brings great challenges. Thus, we have paid increasing attention to the aetiology, management, and treatment of pregnant patients with PH. Hence, by retrospectively analysing the medical records of 154 pregnant patients with PH who were admitted to the Third Affiliated Hospital of Guangzhou Medical University in the past 10 years, we aimed to explore the aetiology and pregnancy outcome in women with PH and provide clinical evidence for the management of such high-risk pregnant women.

## Materials and methods

### Clinical information

**Fig. 1:**
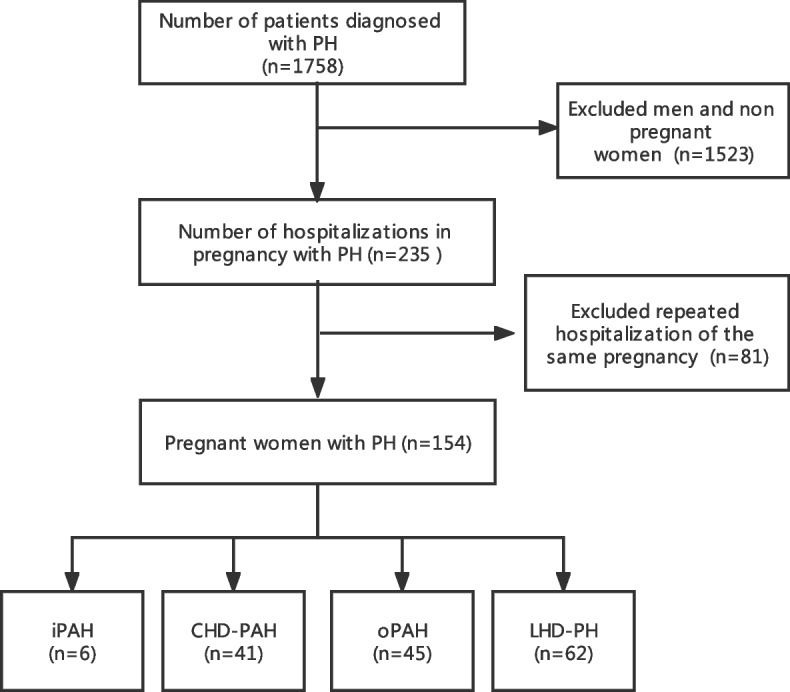
Flow chart of the study. iPAH, idiopathic pulmonary arterial hypertension; CHD-PAH, pulmonary arterial hypertension associated with congenital heart disease; oPAH, pulmonary arterial hypertension associated with other diseases; LHD-PH, pulmonary hypertension caused by left heart disease

This study identified 154 pregnant women with PH who were admitted to the Third Affiliated Hospital of Guangzhou Medical University from January 2011 to December 2020 as the research subjects (Fig. 1). The clinical data, including cardiac function, systolic pulmonary artery pressure (sPAP), regular obstetric examinations, birth method, anaesthesia method, postpartum complications, foetal or neonatal status and other indicators, were analysed. In this study, it was inevitable that some clinical data would be incomplete; missing data were supplemented by telephone follow-up.

### Diagnosis, inclusion and exclusion criteria of PH

According to the diagnostic criteria of the “2015 ESC/ERS Guidelines for the Diagnosis and Treatment of Pulmonary Hypertension”, the haemodynamic diagnostic criteria are that the mean pulmonary artery pressure (mPAP) measured by right heart catheterization at sea level and at rest ≥25 mmHg, pulmonary artery wedge pressure (PAWP) ≤15 mmHg and pulmonary vascular resistance (PVR) > 3 Wood units; the diagnostic criteria for idiopathic pulmonary arterial hypertension (iPAH) should not only meet the PAH criteria diagnosis but also exclude a family history of PAH and all possible secondary factors that may cause PAH [10]. Strict diagnostic criteria should be based on right heart catheterization data. However, considering that the right cardiac catheterization is an invasive examination and the particularity of pregnant women, it is impossible to routinely perform cardiac catheterization to obtain pulmonary artery pressure in pregnant women.

In obstetrics, the sPAP is often indirectly evaluated by the tricuspid regurgitation pressure difference under cardiac colour Doppler ultrasound, and an sPAP ≥36 mmHg (1 mmHg = 0.133 kPa) is used as the diagnostic standard of PH [10]. In this study, the inclusion criteria were as follows: all subjects had undergone cardiac echocardiography, which met the diagnostic criteria of echocardiography, been transferred to the ICU for hemodynamic monitoring during the perinatal period, or had a clinical diagnosis of PH that was confirmed by right cardiac catheterization in the cardiovascular department after delivery [11]. The exclusion criteria were as follows: patients with an elevated sPAP caused by outflow tract obstruction/pulmonary stenosis.

In this study, the timing of pregnancy termination and the mode of delivery of the study subjects were determined by a multidisciplinary team, which comprised experts in obstetrics, critical care medicine, anaesthesiology, and cardiovascular medicine. After discussion, they selected the timing and mode of delivery that was most conducive to the maternal and foetal outcomes based on the level of pulmonary artery pressure, cardiac function, and foetal conditions of the pregnant women.

### PH subgroups

According to the recent expert consensus, PH can be divided on the basis of its aetiology into idiopathic pulmonary arterial hypertension (iPAH), congenital heart disease-related pulmonary arterial hypertension (CHD-PAH), pulmonary arterial hypertension caused by other diseases (oPAH) and PH due to left heart disease (LHD-PH) [2, 10, 12]. Conditions involving LHD-PH include left ventricular systolic or diastolic dysfunction and valve disease. The other diagnoses mentioned in the consensus document were not present in our study population. According to the severity of sPAP, patients with an sPAP of 36–49 mmHg had mild PH, those with an sPAP of 50–69 mmHg had moderate PH, and those with an sPAP ≥70 mmHg had severe PH [8, 11].

### Statistical methods

All analyses were performed using Free Statistics software (version 1.4) (www.freestatistics.tk) and SPSS statistical software (version 25.0) (SPSS Inc., Chicago, IL, USA). Count data are described by frequencies and percentages, and the statistical differences among groups were compared by χ^2^ tests or Fisher’s exact tests. The Shapiro-Wilk (S-W) method was used to test measurement data with a normal distribution; if the data conformed to a normal distribution, which are represented by the mean ± standard deviation (SD), the mean difference among groups was tested by one-way ANOVA, and the LSD-t test was used for pairwise multiple comparisons. If the variance was not uniform, the Dunnett-t test was used. If the measurement data did not conform to a normal distribution, which are expressed by the median (interquartile interval), the mean difference among groups was tested by the rank sum (Kruskal-Wallis H) test, and the Nemenyi method was used for pairwise multiple comparisons. A *P* value of < 0.05 was considered statistically significant (two-sided test).

## Results

### Baseline characteristics

A total of 1758 patients with PH were included in the analysis of the earlier study. After step-by-step exclusion, 154 pregnant women were ultimately identified as the study subjects. (Fig. 1).

In the iPAH group, 6 patients (3.9%) had a median sPAP value of 107.0 mmHg (Q1-Q3 = 78.8–125.5). In the CHD-PAH group, 41 patients (26.6%) had a median sPAP value of 59.0 mmHg (Q1-Q3 = 39.0–92.0). Among them, 25 patients were diagnosed with atrial septal defects, 9 were diagnosed with ventricular septal defects, 4 were diagnosed with patent ductus arteriosus, 1 was diagnosed with atrioventricular septal defects, 1 was diagnosed with congenital single ventricle and 1 was diagnosed with congenital single atrium. In the 54 patients (29.2%) in the oPAH group, the median sPAP value was 40.0 mmHg (Q1-Q3 = 36.0–49.0); 12 women were diagnosed with SLE and 3 women were diagnosed with typical or atypical antiphospholipid antibody syndrome. Among the 62 patients (40.3%) with LHD-PH, the median sPAP value was 50.0 mmHg (Q1-Q3 = 38.2–62.8). There were 38 pregnant women diagnosed with heart valve disease, 6 pregnant women diagnosed with myocardial disease, 15 pregnant women diagnosed with preeclampsia and 3 pregnant women diagnosed with chronic hypertension (Table 1).

**Table 1:** Classification of pregnant women with PH

Aetiology of PH	n	%
**iPAH**	**6**	**3.90%**
**CHD-PAH**	**41**	**26.60%**
Atrial septal defect	25	16.20%
Ventricular septal defect	9	5.80%
Atrioventricular septal defect	1	0.60%
Patent ductus arteriosus	4	2.60%
Congenital single atrium	1	0.60%
Congenital single ventricle	1	0.60%
**oPAH**	**45**	**29.20%**
Systemic lupus erythematosus	12	7.80%
Typical or atypical antiphospholipid antibody syndrome	3	1.90%
Hyperthyroidism	6	3.90%
Gestational diabetes mellitus	10	6.50%
Unknown cause	14	9.10%
**LHD-PH**	**62**	**40.30%**
Heart valve disease	38	25.30%
Cardiomyopathy	6	3.90%
Preeclampsia	15	9.70%
Chronic hypertension	3	1.90%

*iPAH* idiopathic pulmonary arterial hypertension; *CHD-PAH* pulmonary arterial hypertension associated with congenital heart disease; *oPAH* pulmonary arterial hypertension associated with other diseases; *LHD-PH* pulmonary hypertension caused by left heart disease

The ages of the subjects ranged from 21 to 46 years, and their average age was 30.9 ± 5.1 years. The gestational weeks during hospitalization ranged from 6 to 41 weeks, with a median of 35.5 weeks (Q1-Q3 = 31.0–38.0). A total of 129 women (83.8%) had regular obstetric examinations, but there were still 25 women (16.2%) who lacked regular obstetric examinations during pregnancy. There was a statistically significant difference between women who received regular obstetric examinations and those who received no obstetric examinations (*P* <  0.05). Fifty-four pregnant women were transferred to the intensive care unit because of critical conditions, accounting for 35.1% of the total study sample. The iPAH group had the highest intensive care unit (ICU) admission rate (83.3%), followed by the LHD-PH group (37.1%) and then the CHD-PAH group (36.6%). The oPAH group had the lowest ICU admission rate (24.4%), and the difference was statistically significant (*P* <  0.05). The overall hospital length of stay for the pregnant women ranged from 1 to 44 days, with a median value of 9 days (Q1-Q3 = 6.0–13.0). There were 82 patients (53.2%) in the mild PH group, 34 patients (22.1%) in the moderate PH group and 38 patients (24.7%) in the severe PH group (Table 2).

**Table 2:** Baseline characteristics of the participants

Covariates	Total (*n* = 154)	PH groups				*P*-value
		iPAH (*n* = 6)	CHD-PAH (*n* = 41)	oPAH (*n* = 45)	LHD-PH (*n* = 62)	
Age, mean ± SD	30.9 ± 5.1	28.7 ± 4.5	30.6 ± 4.8	30.9 ± 4.6	31.5 ± 5.7	0.539
Gestational week, Median (IQR)	35.5 (31.0, 38.0)	34.5 (30.2, 36.5)	36.0 (30.0, 38.0)	36.0 (29.0, 39.0)	35.0 (32.0, 38.0)	0.958
Primipara, n (%)	99 (64.3)	2 (33.3)	24 (58.5)	28 (62.2)	45 (72.6)	0.175
Multiparous, n (%)	46 (29.9)	4 (66.7)	14 (34.1)	13 (28.9)	15 (24.2)	0.173
sPAP, Median (IQR)	47.0 (37.0, 67.2)	107.0 (78.8, 125.5)	59.0 (39.0, 92.0)	40.0 (36.0, 49.0)	50.0 (38.2, 62.8)	< 0.001
sPAP, n (%)						< 0.001
36–49 mmHg	82 (53.2)	1 (16.7)	16 (39)	35 (77.8)	30 (48.4)	
50–69 mmHg	34 (22.1)	0 (0)	7 (17.1)	8 (17.8)	19 (30.6)	
≥ 70 mmHg	38 (24.7)	5 (83.3)	18 (43.9)	2 (4.4)	13 (21)	
**Place of residence, n (%)**						0.128
City	79 (51.3)	1 (16.7)	19 (46.3)	21 (46.7)	38 (61.3)	
rural	75 (48.7)	5 (83.3)	22 (53.7)	24 (53.3)	24 (38.7)	
**Regular obstetric examinations, n (%)**						0.038
**NO**	25 (16.2)	3 (50)	10 (24.4)	5 (11.1)	7 (11.3)	
**YES**	129 (83.8)	3 (50)	31 (75.6)	40 (88.9)	55 (88.7)	
**NYHA cardiac function classification, n (%)**						< 0.001
I	82 (53.2)	2 (33.3)	16 (39)	36 (80)	28 (45.2)	
II	30 (19.5)	0 (0)	15 (36.6)	2 (4.4)	13 (21)	
III	19 (12.3)	1 (16.7)	9 (22)	0 (0)	9 (14.5)	
IV	23 (14.9)	3 (50)	1 (2.4)	7 (15.6)	12 (19.4)	
Length of stay (days), Median (IQR)	9.0 (6.0, 13.0)	6.5 (2.0, 14.0)	8.0 (5.0, 11.0)	8.0 (5.0, 15.0)	10.0 (7.0, 12.8)	0.279

*NYHA* New York Heart Association. *ICU* Intensive Care Unit

### Pregnancy outcomes

#### Maternal outcomes

In this study, 29 (18.8%) patients delivered vaginally and 108 (70.1%) delivered by caesarean section. The proportion of caesarean sections was significantly higher than that of vaginal deliveries. Caesarean section is the main mode of delivery for pregnant women with PH. Among the patients who terminated their pregnancy by caesarean section, 52 (33.8%) were selected for general anaesthesia, and 56 (36.4%) were selected for intraspinal anaesthesia. In this study, 26 (16.9%) pregnant women had uterine asthenia after delivery, 23 (14.9%) had heart failure during the perinatal period, 8 (5.2%) had multiple organ dysfunction syndrome (MODS), and 1 (0.6%) had cardiac arrest during the perinatal period, but the rescue was timely and effective, saving her life (Table 3).

**Table 3:** Maternal and foetal outcomes

Covariates	PH groups					*P*-value
	Total (*n* = 154)	iPAH (*n* = 6)	CHD-PAH (*n* = 41)	oPAH (*n* = 45)	LHD-PH (*n* = 62)	
**Maternal outcomes**						
**Mode of delivery, n (%)**						0.006
Caesarean section	108 (70.1)	4 (66.7)	31 (75.6)	23 (51.1)	50 (80.6)	
Vaginal delivery	29 (18.8)	2 (33.3)	3 (7.3)	16 (35.6)	8 (12.9)	
**Way of anaesthesia, n (%)**						0.005
General anaesthesia	52 (33.8)	4 (66.7)	11 (26.8)	11 (24.4)	26 (41.9)	
Spinal anaesthesia	56 (36.4)	0 (0)	20 (48.8)	12 (26.7)	24 (38.7)	
**Complications**						
NYHA cardiac function III-IV	42 (27.3)	4 (66.7)	10 (24.4)	7 (15.6)	21 (33.9)	0.026
Admission to ICU, n (%)	54 (35.1)	5 (83.3)	15 (36.6)	11 (24.4)	23 (37.1)	0.032
Postpartum haemorrhage, n (%)	16 (10.4)	1 (16.7)	3 (7.3)	8 (17.8)	4 (6.5)	0.197
Uterine fatigue, n (%)	26 (16.9)	1 (16.7)	5 (12.2)	10 (22.2)	10 (16.1)	0.642
Heart failure, n (%)	23 (14.9)	4 (66.7)	1 (2.4)	6 (13.3)	12 (19.4)	< 0.001
MODS, n (%)	8 (5.2)	3 (50)	1 (2.4)	2 (4.4)	2 (3.2)	0.003
Cardiac arrest, n (%)	1 (0.6)	0 (0)	0 (0)	1 (2.2)	0 (0)	0.607
Maternal death, n (%)	5 (3.2)	3 (50)	1 (2.4)	0 (0)	1 (1.6)	< 0.001
**Foetal outcome**						
Premature infants (< 37 weeks), n (%)	66 (42.9)	4 (66.7)	13 (31.7)	17 (37.8)	32 (51.6)	0.122
(34 ~ 37 weeks), n (%)	30 (19.5)	2 (33.3)	4 (9.8)	7 (15.6)	17 (27.4)	0.088
(< 34 weeks), n (%)	36 (23.4)	2 (33.3)	9 (22)	10 (22.2)	15 (24.2)	0.897
Birth weight of the newborn, mean ± SD	2334.9 ± 814.6	2124.0 ± 837.4	2341.3 ± 799.3	2522.6 ± 974.0	2243.8 ± 718.8	0.374
LBW, n (%)	44 (28.6)	3 (50)	8 (19.5)	7 (15.6)	26 (41.9)	0.004
VLBW, n (%)	20 (13.0)	0 (0)	7 (17.1)	3 (6.7)	10 (16.1)	0.367
ELBW, n (%)	5 (3.2)	1 (16.7)	1 (2.4)	3 (6.7)	0 (0)	0.031
SGA, n (%)	94 (61.0)	4 (66.7)	25 (61)	17 (37.8)	48 (77.4)	< 0.001
Therapeutic abortion	15 (9.7)	0 (0)	7 (17.1)	5 (11.1)	3 (4.8)	0.204
Intrauterine death, n (%)	7 (4.5)	0 (0)	1 (2.4)	5 (11.1)	1 (1.6)	0.132
Missed abortion, n (%)	1 (0.6)	0 (0)	0 (0)	1 (2.2)	0 (0)	0.589
Neonatal death (< 1 week), n (%)	3 (1.9)	0 (0)	1 (2.4)	1 (2.2)	1 (1.6)	0.43

*LBW *low birth weight (> 1500 g, < 2500 g); *VLBW* very low birth weight (1000–1500 g); *ELBW* extremely low birth weight (< 1000 g); *SGA* small for gestational age; *MODS* multiple organ dysfunction syndrome

In this study, 149 patients (96.8%) survived, and 5 patients (3.2%) died. There were no deaths during pregnancy. All 5 women died in the postpartum period. Maternal details are shown in Table 4. None of the 5 women who died had PH diagnosed before pregnancy; all of them were diagnosed with PH during pregnancy and hospitalization. They did not have regular obstetric examinations before admission and did not pay enough attention to their own health management during pregnancy. The New York Heart Association (NYHA) cardiac function classification of the pregnant women who died was IV at admission. The maternal mortality rate was the highest in the iPAH group (3/6, 50%), followed by the CHD-PAH group (1/41, 2.4%) and the LHD-PH group (1/62, 1.6%). Among the six pregnant women in the iPAH group, only 3 improved because they were treated with targeted drugs (Treprostinil) in the perioperative period, which helped dilate pulmonary blood vessels and reduce the cardiac load and pulmonary arterial pressure. However, the other 3 pregnant women in the iPAH group (patients 1, 2, and 4) who died were all in critical condition and transferred from other hospitals. They did not undergo regular prenatal examinations during pregnancy. Due to postpartum haemorrhage complicated with the induction of a PH crisis, even though the patients were under positive rescue, their disease still could not be reversed, and they died. Another case of LHD-PH occurred in a pregnant woman who presented heart valve disease complicated by thrombosis, cerebrovascular embolism, sepsis, etc., and then multiple organ dysfunction. Ultimately, she died of respiratory and circulatory failure on the 6th day after a miscarriage operation; another pregnant woman with CHD-PAH who died (patient 5) suffered from massive haemorrhage and respiratory and circulatory failure after vaginal delivery in a primary hospital. She was in critical condition when transferred from another hospital and died before targeted drug therapy was performed (Table 4).

**Table 4:** Clinical characteristics of the 5 pregnant women who died

	Patient 1	Patient 2	Patient 3	Patient 4	Patient 5
Age (years)	35	32	32	30	25
Gestational weeks	39^+ 1^	34	16	29	30
Causes of disease	iPAH	iPAH	LHD-PH	iPAH	CHD-PAH
PH diagnosed during pregnancy	NO	NO	NO	NO	NO
sPAP (mmHg)	75	152	43	90	148
Parity	G_2_P_2_	G_1_P_1_	G_1_P_0_	G_3_P_2_	G_1_P_1_
Regular prenatal examinations	NO	NO	NO	NO	NO
NYHA cardiac function classification	IV	IV	IV	IV	IV
Complication	Postpartum haemorrhage	Postpartum haemorrhage	Respiratory and circulatory failure	Postpartum haemorrhage	Respiratory and circulatory failure
Foetal outcome	Live birth	Live birth	Iatrogenic abortion	Live birth	Foetal death
Time of maternal death	2 days postpartum	7 days postpartum	6 days postpartum	7 days postpartum	2 days postpartum

**Fig. 2:**
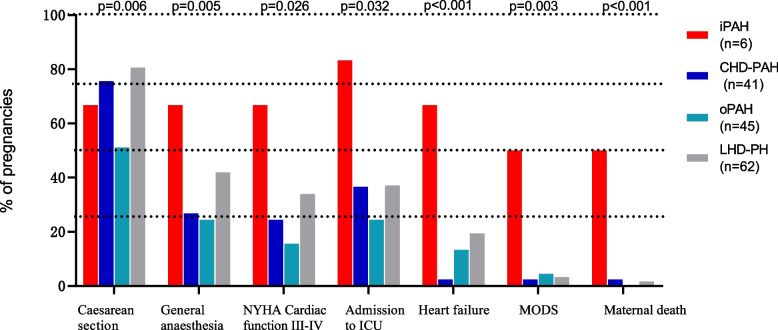
Maternal outcomes in iPAH, CHD-PAH, OPAH, and LHD-PH groups. MODS: multiple organ dysfunction syndrome. NYHA: New York Heart Association. ICU: Intensive Care Unit

Maternal outcomes were compared in the iPAH, CHD-PAH, oPAH, and LHD-PH groups (Table 3 and Fig. 2). There were significant differences in the delivery mode and anaesthesia mode among the four groups (*p* <  0.05). NYHA cardiac function classifications of III-IV, admission to the ICU, heart failure, multiple organ dysfunction syndrome (MODS) and maternal death all showed significant differences (*P* <  0.05). However, there was no significant difference in postpartum haemorrhage (*P* > 0.05).

#### Foetal outcomes

**Fig. 3:**
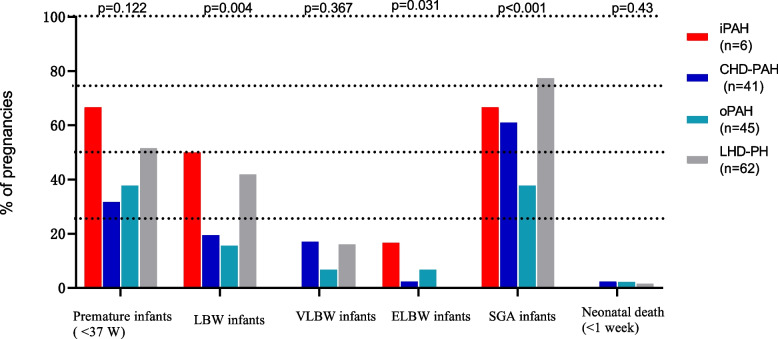
Foetal outcomes in the iPAH, CHD-PAH, OPAH, and LHD-PH groups. LBW: low birth weight (> 1500 g, < 2500 g); VLBW: very low birth weight (1000–1500 g); ELBW: extremely low birth weight (< 1000 g); SGA: small for gestational age; MODS: multiple organ dysfunction syndrome

Among the 154 foetuses of the pregnant patients with PH, 66 (42.9%) were preterm infants (< 37 weeks), including 30 (19.5%) preterm infants born at 34–37-weeks and 36 (23.4%) preterm infants born at < 34 weeks, and the foetal birth weight was 2334.9 ± 814.6 g. There were 44 infants (28.6%) with low birth weight (LBW) (> 1500 g, < 2500 g), 20 infants (13%) with very low birth weight (VLBW) (1000 g–1500 g), 5 infants (3.2%) with extremely low birth weight (ELBW) (< 1000 g), and 94 infants (61.0%) who were small for gestational age (SGA). Three (1.9%) neonates died within 1 week after birth; their mothers were diagnosed with congenital heart disease, rheumatic heart disease and systemic lupus erythematosus. There were 15 cases of therapeutic abortion, accounting for 9.7% of the foetuses. There were 7 cases (4.5%) of intrauterine death, and the average gestational age was (27.7 ± 4.8) weeks; there was 1 case (0.65%) of missed abortion, and the gestational age was 14 weeks (Table 3). Foetal outcomes were compared in the iPAH, CHD-PAH, oPAH, and LHD-PH groups. As shown in Table 3 and Fig. 3, LBW, ELBW and SGA were significantly different (*P* <  0.05) among the groups. However, there were no significant differences in premature infants (< 37 weeks), therapeutic abortion, intrauterine death, and neonatal death (< 1 week) among the four groups (*P* > 0.05).

## Discussion

This was a single-centre retrospective study in southern China, analysing 154 pregnant women regarding maternal, foetal or neonatal complications of PH. Previous studies reported that the median length of survival of PH patients was only 2.8 years [13]. With the application of targeted drug therapy in recent years, the prognosis of PAH patients has been significantly improved. However, some studies have shown that the mortality rate of patients with PH is still as high as 13–18% in the first year [14]. In 2016, Karen Sliwa reported that the mortality rate of pregnant women with pulmonary hypertension within 1 week after delivery was 3.3% [8]. In our study, the 1-week postpartum mortality rate among pregnant women with PH was 3.2%. However, our study was based on data from a single centre and has limitations.

The mechanisms of PH caused by different aetiologies are different, and the treatment and prognosis are also different. Left heart diseases are acquired heart diseases; congenital left heart diseases and acquired heart diseases are the most common causes of PH. Hypoxia in patients with CHD-PAH is a chronic process that is well tolerated by the body, and most of these individuals can adapt to the haemodynamic changes after pregnancy. Left heart diseases are acquired heart diseases; the physiological blood volume increases during pregnancy and is more likely to induce heart failure in patients with LHD-PH, but with timely control of blood volume, most patients can avoid sudden death [12, 15]. Moreover, patients with pre-existing CHD and heart valve disease before pregnancy should focus on health management and regular prenatal examinations during pregnancy. It is easy to achieve early detection and early diagnosis of pregnancy with PH. With early intervention, the prognosis is mostly good. The patients in the oPAH group were generally mildly ill, and the sPAP was usually mild to moderate. For example, the prognosis of patients with systemic lupus erythematosus-related PH is usually better after glucocorticoid pulse therapy and immunosuppressive therapy [16]. In this study, the iPAH group had the worst outcomes, with a mortality rate of 50%. IPAH is caused by multiple factors leading to vasoconstriction, vascular reconstruction and the formation of in situ thrombosis, resulting in haemodynamic and pathophysiological changes, poor cardiac function, and an extremely poor prognosis [17].

Heart failure is a serious complication in pregnant women with PH. Haemodynamic changes in pregnant women are an important reason for the increased risk during pregnancy in this population. First, the blood pressure decreases in early pregnancy but increases in late pregnancy, and this fluctuation of blood pressure increases the risk of adverse pregnancy outcomes; second, with the increase in gestational age, the total blood volume and cardiac output of pregnant women will also gradually increase [13]. The median gestational age of the 5 patients who died was 30 weeks, and the average time of death was 4.8 days after delivery. However, during the 32–34 weeks of pregnancy, the period of delivery and the postpartum period is the period with the most obvious changes in maternal blood volume and haemodynamics, and it is also the period when pregnant women with PH are at high risk of heart failure, pulmonary hypertensive crisis or sudden death [18]. Therefore, for patients with cardiac function classifications of III-IV, our multidisciplinary team suggests that that the pregnancy be terminated at an early stage. Caesarean section should be selected to terminate pregnancy in the middle and late trimesters. Caesarean section can end delivery in a short time, avoid hemodynamic changes caused by prolonged uterine contractions, and reduce the increase in oxygen consumption caused by fatigue and pain.

During normal pregnancy in healthy women, although the blood circulation volume increases, the mean pulmonary arterial pressure (mPAP) does not change significantly, which may be due to pulmonary vasodilation to reduce vascular resistance and thereby adapt to this physiological change [19]. However, in pregnant women with PH, the pulmonary vasodilation capacity decreases, while the pulmonary vascular resistance increases, the pulmonary artery pressure increases, and the blood flow perfusion to the lung decreases, the effect on placental blood flow is unclear [8]. Compared with those of healthy women, foetuses of mothers with CHD are at greater risk of preterm birth and LBW [20]. Foetal and neonatal complications are increased in pregnant women with PH, and the rate of foetal death and neonatal mortality are approximately 10% [8]. Our study showed that the higher the pulmonary arterial pressure was, the higher the rate of LBW infants, and the incidence of LBW infants was higher in the iPAH and LHD-PH groups than in the other two groups. The long-term effects of maternal chronic hypoxia on foetal outcomes are unclear, but the literature reports that LBW infants are at increased risk for future complications such as heart disease, diabetes, and hypertension [21].

## Limitations

This study was a single-centre retrospective investigation conducted in China, with a limited sample of the study populations, and there may be bias in the population representation. Second, since our study population consisted of pregnant women cardiac colour Doppler ultrasonography, but not heart catheterization, was used as a diagnostic tool. This is a common limitation of similar studies.

## Conclusion

There were significant differences in maternal and foetal outcomes in the iPAH, CHD-PAH, oPAH and LHD-PH groups. Maternal mortality was highest in the iPAH group. Therefore, iPAH patients should avoid pregnancy; for high-risk pregnant women who insist on continuing pregnancy, we recommend they see a doctor as soon as possible and standardize treatment. At the same time, we suggest that comprehensive hospitals should establish multidisciplinary assistance teams, including obstetrics, cardiovascular medicine, critical medicine, anaesthesiology and neonatology specialists. Clinicians must balance the pros and cons of maternal and foetal outcomes and choose the most appropriate treatment plan to improve maternal and foetal pregnancy outcomes.

## Data Availability

The raw data supporting this study can be requested from the corresponding author.
